# The BioLink SIG Workshop at ISMB2004

**DOI:** 10.1002/cfg.455

**Published:** 2005

**Authors:** Lynette Hirschman, Christian Blaschke, Alfonso Valencia

**Affiliations:** 1 MITRE Corporation 202 Burlington Road Bedford MA 01730-1420 USA; 2 ALMA Bioinformatica C/Ronda de Poniente, 4 Tres Cantos Madrid 2-C 28760 Spain; 3 Centro Nacional de Biotecnologia Madrid Spain


**Comparative and Functional Genomics**
*Comp Funct Genom* 2005; **6**: 58–60.
Published online in Wiley InterScience (www.interscience.wiley.com). **DOI:** 10.1002/cfg.455

**Conference Editorial**

### The BioLink SIG workshop at ISMB2004

Special Interest Group on Text Data Mining, in association with ISMB 2004, Glasgow, UK; http://www.pdg.cnb.uam.es/BioLink/BioLINK_meetings/2004/index.html

Lynette Hirschman[1]*, Christian Blaschke[2] and Alfonso Valencia[3]
[1] *MITRE Corporation, 202 Burlington Road, Bedford, MA 01730-1420, USA*
[2] *ALMA Bioinformatica, C/Ronda de Poniente, 4, 2-C 28760, Tres Cantos, Madrid, Spain*
[3] *Centro Nacional de Biotecnologia, Madrid, Spain*

**Correspondence to:*
*Lynette Hirschman, MITRE Corporation, 202 Burlington Road, Bedford, MA 01730-1420, USA.*
*E-mail: lynette@mitre.org*

Received: 17 December 2004
Accepted: 21 December 2004

The Special Interest Group (SIG) on Text Mining (or BioLINK — Biological Literature, Information and Knowledge; **http://www.pdg.cnb.uam.es/BioLINK/**) was created to address the need for communication and interchange of ideas in the field of text mining and information extraction applied to biology and biomedicine. Information extraction (IE) is an outgrowth of work in automated natural language processing, which began in the 1950s with work on transformational grammar by Zellig Harris [5,6] and later Noam Chomsky [3,4]. Information extraction technology made rapid progress starting in the late 1980s, thanks to a series of conferences focused on evaluation of IE: the Message Understanding Conferences [1]. There is also a long history of research on applications in medicine. Applications to the medical field focus on two distinct sub-problems: improved access to the medical literature and extraction of information from patient records.

Despite these successes in other fields, natural language processing (NLP) techniques were not introduced in biology until the late 1990s. Even today, there are two distinct groups: on the one hand, researchers with a background in computer science, and on the other hand, their colleagues with a background in the life sciences, with only limited interaction between the two groups. To improve this situation, the BioLINK group holds regular open meetings to bring together researchers developing text data mining tools and related language processing methods to manage the information explosion in the biomedical field. They include invited and contributed papers, with a focus on developing shared infrastructure (tools, corpora, ontologies) and challenge evaluations, in the style of the KDD Challenge Cups [2]. This year, the BioLINK SIG meeting focused on resources and tools for text mining, with special emphasis on the evaluation of these tools. Speakers from the following areas were invited:

- The recent BioCreAtIvE evaluation (Critical Assessment of Information Extraction in Biology).
- TREC Genomics track.
- CASP: Critical Assessment of Techniques for Protein Structure Prediction.
- CAFASP/EVA: Evaluation of automatic structure prediction servers for CAFASP3.
- The BioMINT project.

Copyright © 2005 John Wiley & Sons, Ltd.

### Overview: contributed papers

The contributed papers reflect the importance that is currently given to biological named entity detection in the literature. Four out of the five publications are related to this issue and to the associated issues of resources, infrastructure, and evaluation:

- *A system for identifying named entities in biomedical text*, by Dingare *et al.* The authors present their named entity detection system, which has been applied in two recent assessments (BioCreAtIvE and CoLING BioNLP), and discuss the lessons learned.
- *Protein name tagging guidelines: lessons learned*, by Mani *et al.* Assessments like BioCreAtIvE and CoLING BioNLP need strict guidelines for the named entity annotations used to set up training and test sets distributed to the participants. Mani *et al.* describe the lessons learned in developing a set of guidelines for protein name tagging.
- *A web service for biomedical term look-up*, by Harkema *et al.* The authors present Termino, a large-scale terminological resource for text processing applications that is available as a web service.
- *Towards a semantic lexicon for biological language processing*, by Verspoor. This paper presents an analysis of the UMLS resources, specifically with an eye towards constructing lexical resources suitable for biological language processing.
- *Ontology-based interactive information extraction from scientific abstracts*, by Milward *et al.* This paper describes an ontology-based interactive information extraction framework that enables life scientists to make *ad hoc* queries similar to using a standard search engine but with the advantage of extracting structured information.

### Overview: the invited talks

### Report on the BioCreAtIvE Workshop, Granada, 2004 — Christian Blaschke, Lynette Hirschman, Alexander Yeh, Alfonso Valencia

To formulate common goals, standard datasets and uniform evaluation criteria in biological text mining applications, BioCreAtIvE, a critical assessment of text mining methods, was organized during November/December 2003, inspired by the CASP evaluations.

The first BioCreAtIvE Workshop was held in Granada, Spain, 28–31 March 2004. The goal of the workshop was to provide a set of common challenge evaluation tasks to assess the state of the art for text mining applied to biological problems. The assessment focused on two tasks. The first dealt with extraction of gene or protein names from text, and their mapping into standardized gene identifiers for three model organism databases (fly, mouse, yeast). The second task addressed issues of functional annotation, requiring systems to provide Gene Ontology (GO) annotations for proteins, given full-text articles. Overall, 27 groups participated in the assessment, including 18 for gene/protein name extraction, and nine for the GO functional annotation task.

### Enhancing access to the bibliome: the TREC genomics track — William R. Hersh

The Text Retrieval Conference (TREC) is an annual activity of the information retrieval (IR) research community sponsored by the National Institute for Standards and Technology (NIST). TREC aims to provide a forum for evaluation of IR systems and users. Activity is organized into 'tracks' of common interest, such as question-answering, multi-lingual IR, web searching, interactive retrieval and, as started in 2003, IR in the genomics domain. The genomics track is sustained by a National Science Foundation Information Technology Research grant that provides funding through 2008. Background on the motivation and evolution of the track can be found on the track website (**http://medir.ohsu.edu/~genomics/**). The website also contains an overview paper from the 2003 track as well as the protocol for the 2004 track.

### BioMinT: a database curator's assistant for biomedical text processing — Anne-Lise Veuthey

The goal of the BioMinT project is to develop a generic text mining tool that assists manual database annotation by: (a) interpreting diverse types of query; (b) retrieving relevant documents from the biological literature; (c) extracting the required information; and (d) providing the result as a database slot filler or as a structured report.

Copyright © 2005 John Wiley & Sons, Ltd. 

The development of the BioMinT system has followed a strictly problem-oriented approach. All decisions relative to prototype design have been based on requirements from those who will use the final product in their daily work, i.e. the curators of Swiss-Prot (the knowledgebase component of the UniProt resource) and PRINTS (the protein family fingerprint database), as well as biological researchers.

### CASP: critical assessment of techniques for protein structure prediction — Anna Tramontano

The CASP community-wide experiment critically assesses the state-of-the-art in the prediction of protein structure from sequence and it has been conducted on a 2 year cycle for the last decade, beginning in 1994. The primary goals are to establish the capabilities and limitations of current methods of modelling protein structure from sequence, to determine where progress is being made, to determine where the field is held back by specific bottlenecks, and to compare the results of automatic prediction servers with manually submitted predictions. Methods are assessed on the basis of the analysis of tens of thousands of blind predictions of protein structure submitted by a large number of prediction teams from around the world. CASP provides a forum in which there is a thorough examination of the outcome of the predictions — what went right, what went wrong and, where possible, to provide an understanding of why. For members of the structural biology community not directly involved in structure prediction, the results provide a reasonable guide to the current state of the art. For the prediction community, the results provide a new and sharper sense of direction. Finally, we can begin to measure progress in the field over time.

### EVA: automatic system for the evaluation of structure prediction servers — Burkhard Rost

EVA (**http://www.rostlab.org/eva/**) is a web server for evaluation of the accuracy of automated protein structure prediction methods. The evaluation is updated automatically each week, to cope with the large number of existing prediction servers and the constant changes in the prediction methods. EVA currently assesses servers for secondary structure prediction, contact prediction, comparative protein structure modelling, and threading/fold recognition. Every day, sequences of newly available protein structures in the Protein Data Bank are sent to the servers and their predictions are collected. The predictions are then compared to the experimental structures once a week; the results are published on the EVA web pages. Over time, EVA has accumulated prediction results for a large number of proteins, ranging from hundreds to thousands, depending on the prediction method. This large sample assures that methods are compared reliably. As a result, EVA provides useful information to developers as well as users of prediction methods.

### Acknowledgements

We would like to thank the Program Committee for their careful reviewing: Luc Dehaspe, Robert Gaizauskas, William Hersh, Karin Verspoor and Alexander Yeh. We would also like to thank the invited speakers, whose workshop abstracts appear in this overview. This paper reports on work done in part at the MITRE Corporation under support from the National Science Foundation (Grant No. EIA-0326404).

### References

1. Hirschman L. 1998. The evolution of evaluation: lessons from the message understanding conferences. *Comput Speech Langu* **12**: 281–305; **http://www.itl.nist.gov/iaui/894.02/related projects/muc**.
2. Yeh A, Hirschman L, Morgan A. 2003. Evaluation of text data mining for database curation: lessons learned from the KDD Challenge Cup. *Bioinformatics* **19**: 331–339.
3. Chomsky N. 1956. *Syntactic Structures*. Mouton: The Hague and Paris. .
4. Chomsky N. 1965. *Aspects of the Theory of Syntax*. MIT Press: Cambridge, MA.
5. Harris Z. 1952. Discourse analysis. *Language* **28**: 18–23.
6. Harris Z. 1957. Co-occurrence and transformation in linguistic structure. *Language* **33**(3): 283–340.

Copyright © 2005 John Wiley & Sons, Ltd.

